# P02-012 - HAIDS in practice of Russian rheumatologist

**DOI:** 10.1186/1546-0096-11-S1-A119

**Published:** 2013-11-08

**Authors:** E Fedorov, S Salugina, N Kuzmina

**Affiliations:** 1 Pediatric Department, Research Institute of Rheumatology under the RAMS, Moscow, Russian Federation

## Introduction

Human autoinflammatory diseases (HAIDS) are defined as illnesses caused by primary disfunction of the innate immune system. This diseases had different prevalence in different ethnic groups.

## Objectives

To determine the spectrum of HAIDS in the practice of pediatric rheumatologist on the results of appealability to the Federal rheumatologic center.

## Methods

This study included patients who applied to the Federal Rheumatologic Center in 2009 - 2012 years for diagnosis adjustment because of fever of unknown origin. All patients were submitted to routine rheumatology examination and HLA-I-typing, and molecular genetic testing.

## Results

42 children with HAIDS in age between 2 and 17 years were identified in 3 years. Age of onset ranged from 0 to 16 years, on an average 5.1 years old. The average age at diagnosis was 8.2 years old. The following diseases were identified: Behcet's disease (BD) - 16 children (male/female (M/F) - 12/4); Cryopyrin-Associated Periodic Syndromes (CAPS): Muckle-Wells syndrome - 4 (M/F - 0/4 ); CINCA/NOMID syndrome - 2 (M/F - 2/0); Familial Mediterranean fever (periodical disease) (FMF) - 7 (M/F - 1/6); PFAPA syndrome - 4 (M/F - 2/2), chronic recurrent multifocal (nonbacterial) osteomyelitis (CRMO) - 3 (M/F - 0/3), hyper-IgD syndrome/mevalonate kinase deficiency - 2 (M/F -0/2), undifferentiated HAIDS - 4 (M/F = 2/2). Patient’s ethnicity with BD: peoples of the North Caucasus - 3, Tatars - 4, Uzbeks - 1, Azerbaijanians - 1, Russians - 5, Ukrainians- 1, ethnic Germans- 1. Among the patients with FMF: Armenians- 4, Azerbaijanians- 2. Russian patients dominated for other nosologies. HLA-B51 antigen was detected in 8 (50%) patients with BD. For all patients with FMF, CAPS and for one patient with hyper-IgD syndrome the diagnoses were confirmed by genotyping.

## Conclusion

The pediatric rheumatologist can meet in his practice patients with various HAIDS. The most frequent in our Russian Federal rheumatologic center were BD and FMF. For these diseases the prevalence of certain ethnic groups was identified. The patients with CAPS are the most similar in clinical laboratory performance to rheumatic diseases and they need differential diagnosis of systemic juvenile arthritis and early prescription of targeted therapy (IL-1 inhibitors) that significantly changes the initial poor prognosis.

## Disclosure of interest

None declared.

